# Cupulin Is a Zona Pellucida-Like Domain Protein and Major Component of the Cupula from the Inner Ear

**DOI:** 10.1371/journal.pone.0111917

**Published:** 2014-11-04

**Authors:** Jens Dernedde, Christoph Weise, Eva-Christina Müller, Akira Hagiwara, Sebastian Bachmann, Mamoru Suzuki, Werner Reutter, Rudolf Tauber, Hans Scherer

**Affiliations:** 1 Institut für Laboratoriumsmedizin, Klinische Chemie und Pathobiochemie, Charité -Universitätsmedizin Berlin, Berlin, Germany; 2 Institut für Chemie und Biochemie, Freie Universität Berlin, Berlin, Germany; 3 Max Delbrück Center for Molecular Medicine, Berlin-Buch, Germany; 4 Department of Otolaryngology, Tokyo Medical University, Shinjuku-ku, Tokyo, Japan; 5 Institut für Vegetative Anatomie, Charité - Universitätsmedizin Berlin, Berlin, Germany; 6 Klinik für Oto-Rhino-Laryngologie, Charité - Universitätsmedizin Berlin, Berlin, Germany; Universita' di Padova, Italy

## Abstract

The extracellular membranes of the inner ear are essential constituents to maintain sensory functions, the cupula for sensing torsional movements of the head, the otoconial membrane for sensing linear movements and accelerations like gravity, and the tectorial membrane in the cochlea for hearing. So far a number of structural proteins have been described, but for the gelatinous cupula precise data are missing. Here, we describe for the first time a major proteinogenic component of the cupula structure with an apparent molecular mass of 45 kDa from salmon. Analyses of respective peptides revealed highly conserved amino-acid sequences with identity to zona pellucida-like domain proteins. Immunohistochemistry studies localized the protein in the ampulla of the inner ear from salmon and according to its anatomical appearance we identified this glycoprotein as Cupulin. Future research on structure and function of zona pellucida-like domain proteins will enhance our knowledge of inner ear diseases, like sudden loss of vestibular function and other disturbances.

## Introduction

**Figure 1:**
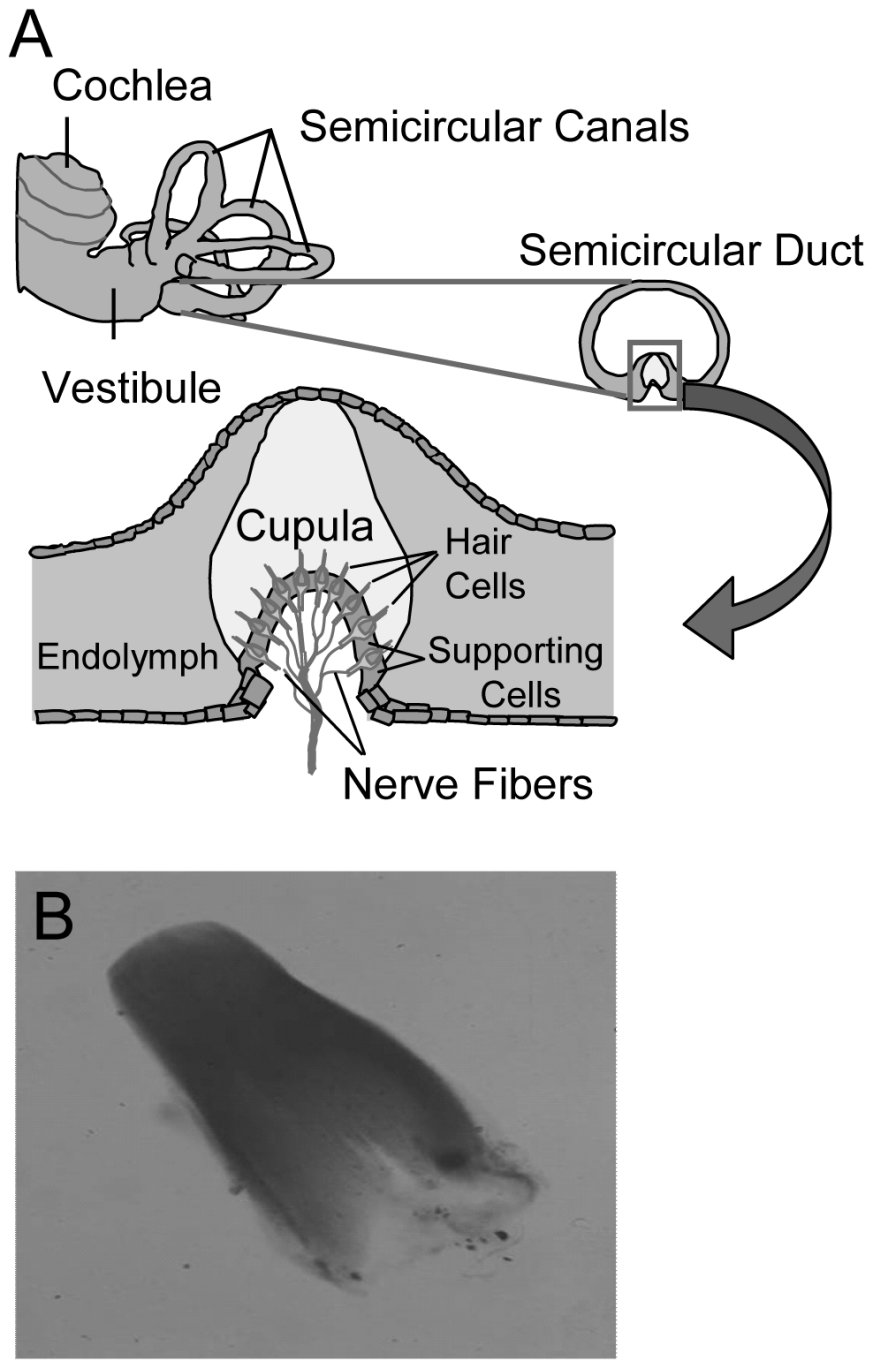
The cupula. **A**, localization of the cupula in the inner ear. **B**, dissected cupula from salmon stained with Evans blue.

The vestibular organ of vertebrates has five mechanical sensors that convert acceleration into electrical signals. They are located in the labyrinth organ of the inner ear. Three of them function as membranes (cupulae) in a liquid-filled cavity (Fig. 1A). The sensors are located in a widened part (ampulla) of a fluid filled ring system, the semicircular canals. The cupulae are fixed at the roof of the ampulla and ride on a barrel-like structure, the crista ampullaris. Kino- and stereocilia growing out from the top of hair cells connect the gelatinous cupula with the underlying neuroepithelium. A torsional acceleration of the head leads to a counter rotation of the fluid resulting in a deflection of the cupula and thereby in a stimulation of the hair cells [1]–[3]. Shearing of tip-links between the hairs opens mechanosensitive ion channels. The result is a potassium influx into the cells which causes a generator potential and in the afferent bipolar nerve an alteration of the action potential rate. A detachment of the cupula from the roof or a leak in the membrane impedes stimulation [4], [5].

In humans suffering from a sudden loss of vestibular function, a malfunction in the ampulla is considered to be a possible explanation. Experiments in pigeons [3], [4] have demonstrated that the mechanical detachment of the cupula from the roof of the ampulla results in the clinical picture of a vestibular loss of function. Furthermore a membrane leak could develop if the structural integrity of the cupula is compromised, e.g. by a lack of structural material production, or by an elevation of the ampulla roof caused by increased pressure of incoming liquid [6].

To gain further knowledge on the origin of sudden loss of vestibular function, we started to analyze the cupula material from salmon and chicken. Goodyear and Richardson [7] have compared the protein composition of acellular matrices of the inner ear, the tectorial and otoconial membranes and the cupula. In the mouse inner ear, α- and β-Tectorin are the major components of the tectorial and otoconial membranes, but are missing in the cupula [7], [8]. Although Otogelin, a 313 kDa protein related to mucins, was found in all acellular structures of the inner ear in mice [7], in otogelin-null mutant the cupula is still present, but detached from the crista ampullaris [9]. Therefore, a so far unknown structural component must be responsible to build the macromolecular cupula structure. Goodyear and Richardson [7] postulated the existence of this structural protein and suggested the name “Cupulin”.

In order to identify this missing component of the cupula, we investigated the inner ear from salmon and chicken. These animals were selected because their vestibular organs are relatively easily accessible. In birds the bony layer of the vestibular organ is very thin and can be removed carefully giving access to the membranous structures. In addition, in fish the vestibular organ is not embedded in bone at all.

## Methods and Chemicals

All chemicals were from Sigma-Aldrich (München, Germany) if not otherwise stated.

### Preparation of cupulae from salmon and chicken

The heads of commercially slaughtered salmon were opened. After suction of the brain, the free endocranial part of the vertical canal was cut and the labyrinth was removed carefully without touching the ampullas. The labyrinth was immediately immerged in artificial endolymph (126 mM KCl, 1 mM NaCl, 25 mM KHCO_3_, 0.025 mM MgCl_2_, 0.025 mM CaCl_2_, 1.4 mM K_2_HPO_4_, 25 mM mannitol, pH 7.4), as described previously by Marcus et al. [10]. For asservation of the salmon cupulae the semicircular canal was cut 3 mm away from the ampulla. With a micropipette the canal was filled on the side of the ampulla with Evans-blue to stain the cupulae for better visualization. Subsequently the canal was cut again just at the site where it enters the ampulla. By an additional longitudinal short cut on the roof, the ampulla was opened. Small movements of the specimen with micro-forceps on both sides detached the cupula. The cupula has almost the same specific weight as the endolymph and does therefore not sink. This effect and its shape allowed distinguishing the cupula from various parts of the specimen, formed during preparation. As long as the acellular cupula was kept in endolymph it did not change either its form or size.

For preparation of cupulae from commercially slaughtered chicken the heads were fixed in an upright position. After removal of the bone of the posterior lateral portion of the head, the semicircular canals and the ampullas were identified. The very thin bony layer was removed with needles and micro-forceps. The labyrinth was removed and stored in artificial endolymph solution. Further preparation steps were identical to those described for the salmon preparation.

### Trypsin digestion and mass-spectrometric analyses

Crude cupula material from salmon and chicken was dissolved in denaturing 2 x SDS sample buffer and boiled for 5 min. Remained debris was removed by centrifugation (14,000×g, 10 min). Soluble extract was separated by SDS-PAGE under reducing conditions and the gel was stained with Coomassie Brilliant Blue. The dominant 45 kDa band was excised from the gel with a scalpel and cut into small 1 mm gel cubes.

Peptides were obtained by trypsin in-gel digestion as described previously [11] and peptide masses were analysed by matrix-assisted laser desorption ionization-time of flight mass spectrometry (MALDI-TOF-MS) using an Ultraflex-II TOF/TOF instrument (Bruker Daltonics, Bremen, Germany) equipped with a 200 Hz solid-state Smart beam laser. The mass spectrometer was operated in the positive reflector mode. Mass spectra were acquired over an m/z range of 600–4,000.

α-cyano-4-hydroxycinnamic acid (CHCA) was used as the matrix and protein digest samples were spotted using the dried-droplet technique. MS/MS spectra of selected peptides were acquired in the LIFT mode [12].

Database searches were performed using Mascot (Matrix Science Ltd., http://www.matrixscience.com). Mass tolerance was typically set at ±75 ppm and we allowed for one missed cleavage. Annotation of the MS/MS spectra was done manually.

### Data analysis

By using the Basic Local Aligment Search Tool BLASTP [13] peptide sequences were screened for similarity in the protein database to assign the protein. For comparison of protein sequence data we used the ClustalW2 program from the European Bioinformatics Institute, EBI [14]. For the detection of the signal peptide we applied the SignalP algorithm [15], the transmembrane domain was predicted by the TMHMM 2.0 software [16]. The protein sequence was further screened for potential *N*-glycosylation sites with the program NetNGlyc 1.0 (Center for Biological Sequence Analysis, Technical University of Denmark).

### Zona pellucida-like domain protein specific peptide antibodies and Western blotting

Peptide-specific antibodies were obtained by standard immunization of guinea pigs with a mixture of two synthetic peptides linked to the KLH antigen (Pineda, Berlin, Germany). The peptide sequences were: P1: NH2-**C**DANFHSRFPAERDI, and P2: NH_2_-VKHKNQKMS TVFLH**C** respectively. Cysteine residues (**C**) were added to the sequence to achieve further peptide coupling. Serum was prepared and the total IgG fraction was first isolated by affinity chromatography on protein A sepharose (GE-Healthcare, München, Germany). The peptide specific antibodies were then purified by peptide affinity chromatography. Therefore 1 mg of both peptides was coupled to 2 ml of thiol sepharose, according to the manufacturers' description (GE-Healthcare, München, Germany). Reactivity to crude cupula preparations was analysed by Western blotting at a 1∶10,000 dilution of purified peptide-antibodies (0.7 mg/ml). The secondary peroxidase-labelled anti-guinea pig antibody was from Dianova (Hamburg, Germany). For blot development the Amersham ECL Western Blotting System Kit form GE Healthcare (München, Germany) was applied.

### *N*-Deglycosylation Analysis

Crude cupula material was first boiled for 5 min in a 1% SDS, 1% β-mercaptoethanol solution, next diluted to a final concentration of 0.1% SDS in 20 mM sodium phosphate, pH 7.4, 1% Nonidet P-40, and digested with peptide:*N*-glycosidase F (PNGase F, New England Biolabs GmbH, Frankfurt, Germany) for 2 hours at 37°C. A typical analytical sample contained 2–3 cupulae and was digested with 0.2 µl enzyme (100 NEB units) in a final reaction volume of 20 µl. Deglycosylation of samples was demonstrated by SDS-PAGE and subsequent protein staining.

### Histochemistry

The vestibular organ of salmon heads was removed from the cerebral cavity and kept in a fixation solution (4% formaldehyde) for 30 min. The ampullas were separated from the stony otoliths and the specimens were embedded in paraffin. Cross-sections of the ampullas from salmon were alternating prepared for either HE-staining or immunohistology with anti-zona pellucida-like domain protein antibodies (1∶100). The secondary antibody was a commercial PE-labeled anti-guinea pig antibody (Dianova, Hamburg, Germany)

## Results

**Figure 2:**
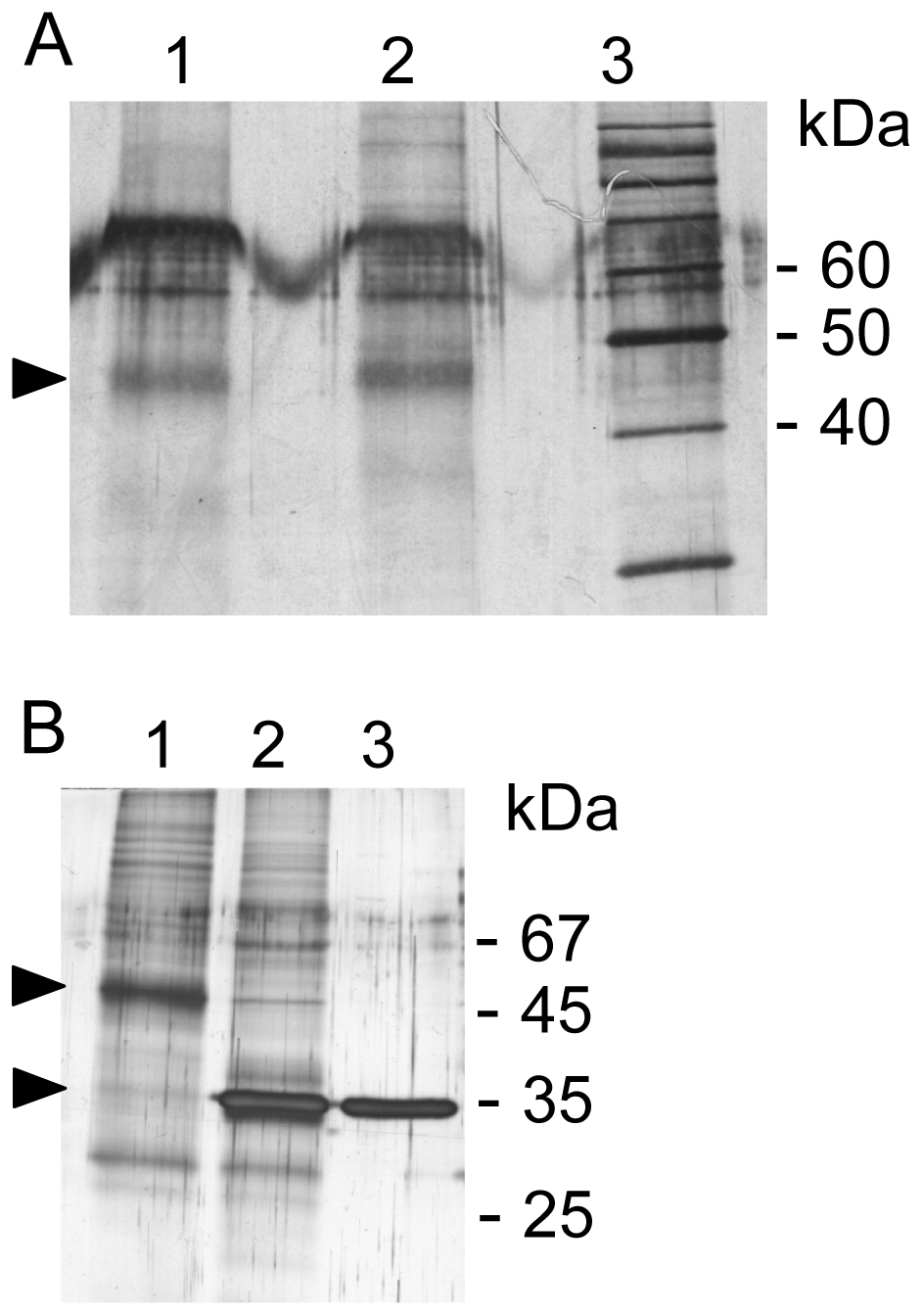
Visualization of cupula proteins. **A**, crude extracts from isolated cupulae from salmon, (lane 1) and chicken (lane 2) were separated on a 12% SDS-PAGE under reducing conditions and silver stained. The arrowhead highlights a dominant protein (∼45 kDa) chosen for further analyses. Lane 3, marker proteins. In the 60 kDa range additional yet unidentified protein components are visible. **B**, deglycosylation of salmon cupula protein extract. Lane 1, cupula extract untreated; lane 2, cupula extract+PNGase F (100 NEB units), lane 3, PNGase F control (500 NEB units). Arrowheads indicate molecular weight shift of the 45 kDa protein due to the *N*-deglycosylation.

The cupula is a jelly-like extracellular matrix of the inner ear and part of the sensor system that measures torsional accelerations (Fig. 1). When we started to analyze the cupula protein composition from salmon and chicken by gel electrophoresis, a comparable protein pattern with ∼10–15 bands, depending on the quality of sample preparation was detected for both organisms (Fig. 2). The existence of a dominant protein that constitutes the cupula structure was predicted by Goodyear and Richardson [7]. Here we identified a prominent fuzzy band in the range of approximately 45 kDa after sample separation from salmon and chicken under denaturing and reducing conditions (Fig. 2A). Although the protein was always visible, the distinctness varied between different preparations. As extracellular matrices usually consist of glycosylated proteins we treated the cupula sample from salmon with the *N*-glycosidase PNGase F. Indeed the size of the dominant 45 kDa cupula protein was reduced by ∼11 kDa to a size of 34 kDa (Fig. 2 A,B). The PNGase F control (lane 3) migrated at the same position, but here we loaded the fivefold quantity of enzyme compared to the amount in lane 2 to visualize the protein. From the disappearance of the 45 kDa band and the intense staining at 34 kDa we concluded that the deglycosylated salmon protein and PNGase F run at the same position. Faintly stained protein bands in lanes 1 and 2 that migrate approximately 20 kDa below the glycosylated or deglycosylated protein, may reflect immunoreactive degradation products (Fig. 2B).

**Figure 3:**
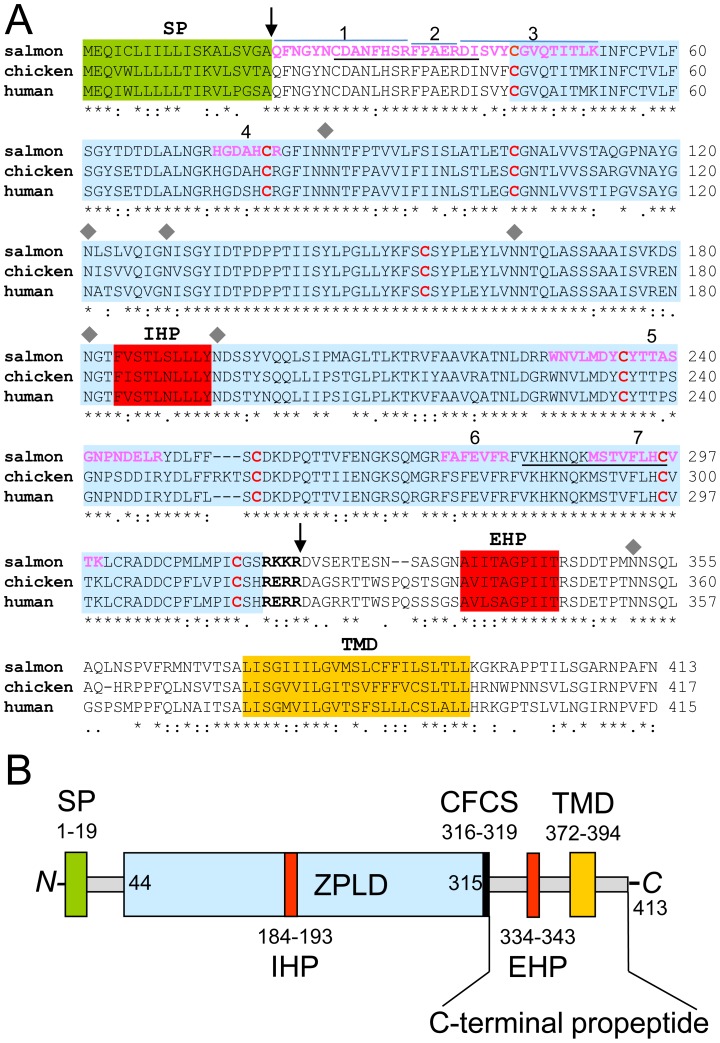
Zona pellucida-like domain protein homology and structure. **A**, protein sequences from UniProt database: salmon: C0H9B6; chicken: E1C8E6; human: Q8TCW7 were aligned by applying the ClustalW2 program. Asteriks (★) marked below the sequence highlight conserved amino-acids (∼70%) between the three organisms. Conserved cysteine residues 1–8 that constitute the zona pellucida-like domain (blue box) are shown in red letters. Arrows mark the mature protein after cleavage of the *N*-terminal signal sequence (SP, green box) predicted by the SignalP algorithm and the *C*-terminal furin cleavage site (CFCS, black and bold letters). IHP and EHP (red boxes) show the potential internal and external hydrophobic patches of the zona pellucida-like-domain (ZLPD). The transmembrane domain (TMD, orange box) was predicted by the TMHMM 2.0 software. In pink and bold are highlighted the peptides 1–7, identified by mass spectrometry. Individual peptides 1, 2 and 3 are highlighted with blue lines. Underlined peptide sequences were used for immunization. Grey diamonds indicate the asparagine residue of potential *N*-glycosylation sites (NXS/T) determined with the program NetNGlyc 1.0. **B**, scheme of zona pellucida-like domain protein structure.

To identify specific peptide sequences from the salmon protein, gel electrophoresis was performed, the 45 kDa band was cut-out, and the protein was trypsinized and further analyzed by mass spectrometry. The peptide mass fingerprint analysis with annotated peptide sequences is shown in Fig. S1 and exemplarily a detailed MS/MS spectrum for one peptide is presented in Fig. S2. Database searches revealed identity to several predicted open reading frames of zona pellucida-like domain proteins. Here, to the best of our knowledge we identified for the first time corresponding peptide sequences from a zona pellucida-like domain protein (Table 1). The overall match of seven identified peptides with the predicted sequences from salmon, chicken, and human origin is convincing (Fig. 3A) and covers about 26% of the mature extracellular protein ranging from amino-acids 21 to 319 (Fig. 3) With peptide 1 we most probably identified the *N*-terminus of the secreted zona pellucida-like domain protein. Cleavage of the signal peptide between amino-acids A_19_ and Q_20_ is predicted by the SignalP algorithm (data not shown). Furthermore the conversion of glutamine to pyroglumate (Table 1) argues for the *N*-terminal position, where spontaneous intramolecular cyclization can occur. In addition it is interesting to note, that we observed one difference to the published sequence (C0H9B6) from the salmon zona pellucida-like domain protein. Instead of the uncharged asparagine (N_22_) we identified the acidic aspartic acid residue (D_22_) in peptides 1 and 1^a^. Further amino-acid changes might be explained by the interindividual variations due to our randomly pooled material from wild and farm-raised salmon from diverse origin. In detail, in addition to F_30_ present in peptide 1 a substitution to Y_30_ was identified in peptide 1^a^. Further in addition to the correctly matching sequence from peptide 5, two variations were identified compared to the database entry within peptide 5^a^, where V_229_ was changed to I_229_ and A_239_ to P_239_. In each case the amino-acid substitution was conservative and hydrophobic apolar amino-acids were used.

**Table 1:** Peptide sequences obtained from the 45 kDa gel band from salmon.

Peptide No.	Mass observed (g/mol)	Mass calculated (g/mol)	Peptide sequences
1	1713.71	1712.71	pyroQF**D**GYNCDANFHSR (+cam)
1^a^	1729.72	1728.71	pyroQF**D**GYNCDAN**Y**HSR (+cam)
2	619.26	618.31	FPAER
3	1596.87	1595.80	DISVYCGVQTITLK (+cam)
4	852.35	851.32	HGDAHCR (+cam)
5	2519.13	2518.09	WNVLMDYCYTTASGNPNDELR (+cam)
5^a^	2559.16	2558.12	WN**I**LMDYCYTT**P**SGNPNDELR (+cam)
6	915.47	914.47	FAFEVFR
6*	972.50	971.47	FAFEVFR (+cam)
7	1322.70	1321.65	MSTVFLHCVTK (+cam)
7*	1338.70	1337.65	MSTVFLHCVTK (+cam+ox)

Data base searches were performed using Mascot and annotation of the MS/MS spectra was done manually. Amino-acid residues that differ from the published salmon sequence (C0H9B6) are bold. Amino-acid modifications: pyroQ, pyroglutamate, (delta mass: −17); ox, oxidized methionine (delta mass: +16); cam, carbamidomethyl, (delta mass: +57).

a peptide with additional amino-acid exchange.

*peptide with additional modification.

The alignment of protein sequences from salmon (C0H9B6), chicken (E1C8E6) and human (Q8TCW7) was performed with the ClustaW algorithm. Overall, the zona pellucida-like domain proteins are highly conserved from fish to human, with a sequence identity of 72%. According to the nomenclature by Bork and Sander [17] which is based on the positioning of conserved amino-acids, i.e. structuring cysteine residues, the protein contains a zona pellucida-like domain (Figure 3), but exhibits only minor amino-acid sequence identity to the zona pellucida (ZP) domain, when compared to the well-studied murine sperm receptor mZP3 (data not shown). On the other hand, the conserved regions of zona pellucida-like domain protein and the ZP of mZP3 imply a similar structure and therefore hint at comparable function of both proteins as described [18]–[22].

When we compared the salmon zona pellucida-like protein to the zona pellucida protein mZP3 following similarities were obvious: i) an *N*-terminal signal peptide directing the protein to the endoplasmatic reticulum (ER) and Golgi apparatus for posttranslational modification, ii) for the zona pellucida-like domain we also predict an *N*-terminal internal hydrophobic patch (IHP) and *C*-terminal external hydrophobic patch (EHP) as demonstrated for mZP3 [19], iii) a consensus furin cleavage site (CFCS) that separates IHP from EHP and enables the extracellular delivery of the mature protein and its polymerization, iv) a transmembrane domain necessary for initial anchoring at the cell membrane.

The zona pellucida-like domain protein from salmon is probably modified after translation. Initially the protein consists of 413 amino-acids (aa) and has a calculated molecular mass of 45.2 kDa. Cleavage of the signal peptide and further processing at the C-terminal furin cleavage site (CFCS), could deliver the mature secreted protein consisting of 299 aa with a molecular mass of 33.4 kDa. As described above, separation of extracted cupula material by SDS-PAGE displayed a dominant fuzzy protein band at 45 kDa (Fig. 2, lane 1). After deglycosylation with PNGase F, the 45 kDa band disappeared and a new band appeared at about 34 kDa (Fig. 2, lane 2), which is consistent with the calculated molecular mass of the mature protein. The difference of 11 kDa can therefore be attributed to posttranslational modification by 3 to 4 *N*-glycan chains depending on their individual structure. In Fig. 3 A potential *N*-glycosylation sites are depicted.

**Figure 4:**
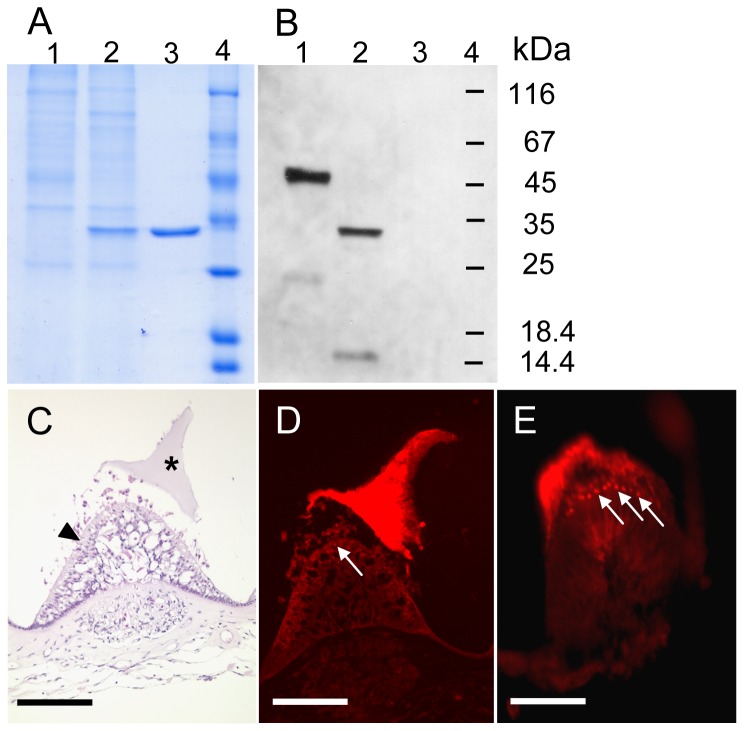
Immunodetection of zona pellucida-like domain protein from salmon samples. **A**, Coomassie stained SDS-polyacrylamide gel and B, corresponding Western blot of separated crude cupula extracts. Lane 1, untreated sample, lane 2, PNGase F treated sample with faster migration of zona pellucida-like domain protein. **C**, HE stained inner ear cross section, asterisk marks the cupula and the arrowhead the subcupulary region with sensory and supporting cells. **D**, immunostaining of inner ear cross section, the arrow probably indicates staining of supporting cells which produce the zona pellucida-like domain protein. This is even more pronounced in **E**.

To further characterize the protein, antibodies were generated from two different peptide sequences. Antibodies were peptide affinity purified from serum of guinea pig and rabbit. As expected the antibodies recognized the 45 kDa and the 33 kDa deglycosylated protein band (Fig. 4B). So it is obvious that the zona pellucida-like domain protein is one major structural protein of the complex cupula structure. Further, we analyzed the protein expression on histological sections of the inner ear in the region of the ampulla. In Fig. 4C the hematoxylin-stained tissue shows the detached and shrunken cupula which sits on top of the neuroepithelium *in vivo* connected with the hair cells. In Fig. 4D the cupula is intensely stained by the zona pellucida-like domain protein-specific antibodies and in the neuroepithelium faint red-stained dots are visible, which are more pronounced in Fig. 4E. We assume that the red dots represent protein-loaded vesicles that derive from the zona pellucida-like domain protein-producing supporting cells (Fig. S3).

## Discussion

Cupulin is the missing link of cupula structural material predicted by Goodyear and Richardson [7]. Here we show for the first time that this zona pellucida-like domain protein from salmon is a major structural component of the ampullary cupula which senses torsional accelerations of the head. A similar protein profile (Fig. 2A) of salmon and chicken samples indicates a common cupula architecture, but this has to be proven in the future in more detail.

Zona pellucida-like domain proteins are highly conserved. The comparison of the deduced amino-acid sequences from salmon, chicken and human reveals a high overall identity (Fig. 3A). A minor identity to ZP proteins, e.g. murine ZP3, is predominantly based on the conserved arrangement of cysteine residues. Nevertheless, both proteins are synthesized as precursor polypeptides with an *N*-terminal signal sequence and a *C*-terminal propeptide that contains the consensus furin cleavage site (CSFS), the external hydrophobic patch (EHP), a transmembrane region and a short cytoplasmic tail (Fig. 3B). To avoid ZP polymerisation, it is assumed for ZP3 that the EHP binds to the internal hydrophobic patch (IHP) during intracellular vesicular transport [21], [22]. After fusion of secreted ZP3-containing vesicles with the cellular membrane the propeptide is thereafter released by cleavage of the CSFS enabling the mature protein to start homopolymerisation [19], [23]. We propose a similar mechanism for zona pellucida-like domain protein assembly. It is likely that the release of the mature secreted protein to the ECM triggers polymerization and is a prerequisite for assembly of a macromolecular structure in the inner ear, the cupula. Here we have described a main building material of the salmon cupula, the zona pellucida-like domain protein at the molecular level. Protein expression takes place in the supporting cells of the crista ampullaris which surround the sensory hair cells (Fig. 1 and Fig. S3). The synthesis must be strictly controlled otherwise sensing of torsional accelerations could not be accurately measured. A sudden loss of vestibular function may originate from a failure of zona pellucida-like protein synthesis. In agreement with this assumption, treatment with antibiotics that block protein synthesis leads to a loss of vestibular function and to an atrophy of the cupula structure [24], [25].

## Supporting Information

Figure S1 MS/MS spectrum of trypsin digested 45 kDa salmon protein. Peptide mass fingerprint with annotated peptide sequences. The protein was identified as zona pellucida-like protein (C0H9B6). Peptide numbers correspond to numbers given in Figure 3A.
(TIF)

Figure S2 Detailed MS/MS spectrum of peptide: FAFEVFR (peptide 6). b and y ion series with inserted fragment ion table.
(TIF)

Figure S3 Electron microscope image of sensory tissue below the cupula. Sensory cells (white arrows) with hairbundles (black arrows) are shown, adjacent to supporting cells (asterix).
(TIF)
