# Supplementary figures and images for: Cupulin Is a Zona Pellucida-Like Domain Protein and Major Component of the Cupula from the Inner Ear
Source: PLoS One. 2014 Nov 4;9(11):e111917. doi: 10.1371/journal.pone.0111917 (PMC4219815; doi:10.1371/journal.pone.0111917)

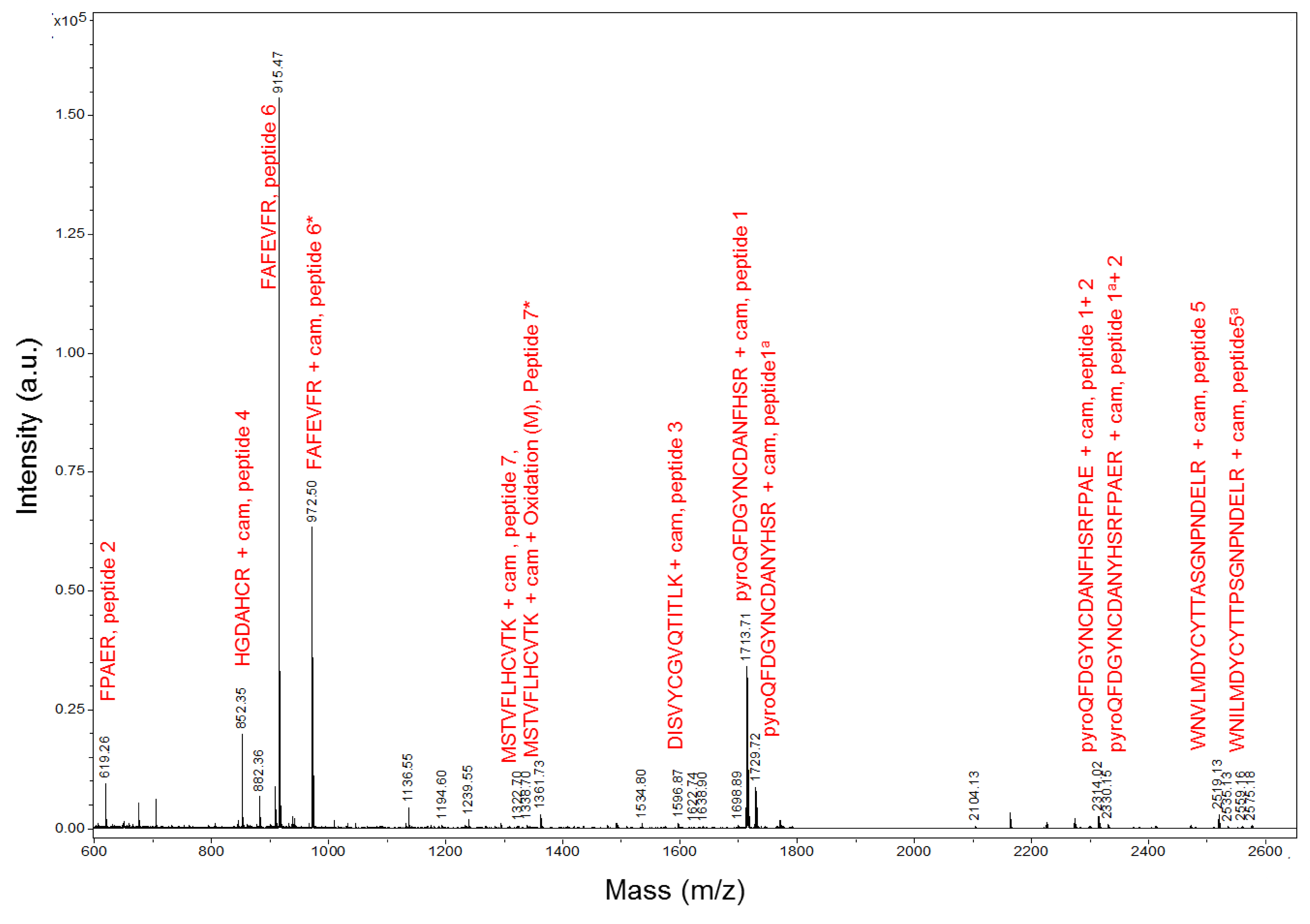

Supplement: Figure S1 — MS/MS spectrum of trypsin digested 45 kDa salmon protein. Peptide mass fingerprint with annotated peptide sequences. The protein was identified as zona pellucida-like protein (C0H9B6). Peptide numbers correspond to numbers given in Figure 3A. (TIF) [file pone.0111917.s001.tif]

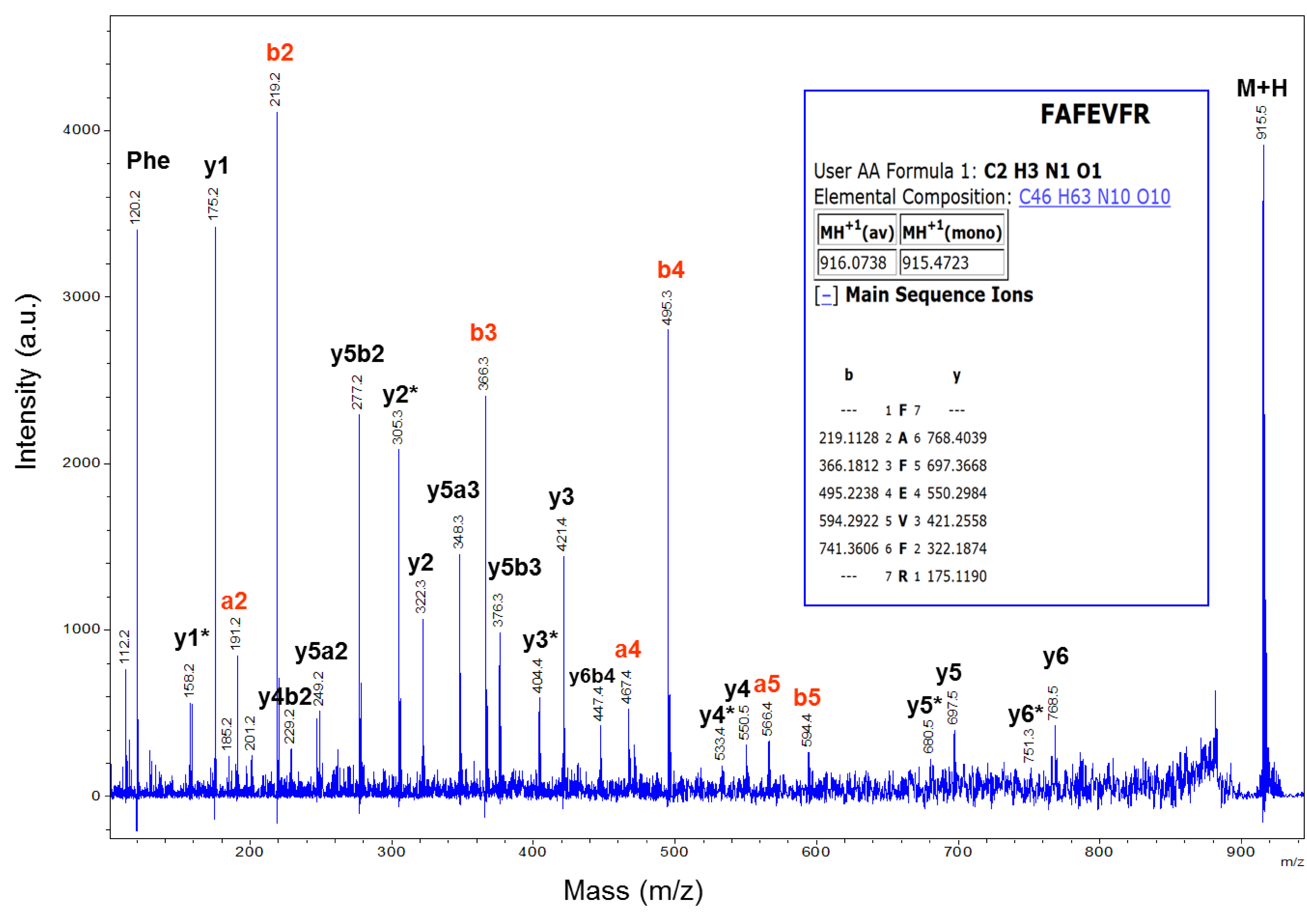

Supplement: Figure S2 — Detailed MS/MS spectrum of peptide: FAFEVFR (peptide 6). b and y ion series with inserted fragment ion table. (TIF) [file pone.0111917.s002.tif]

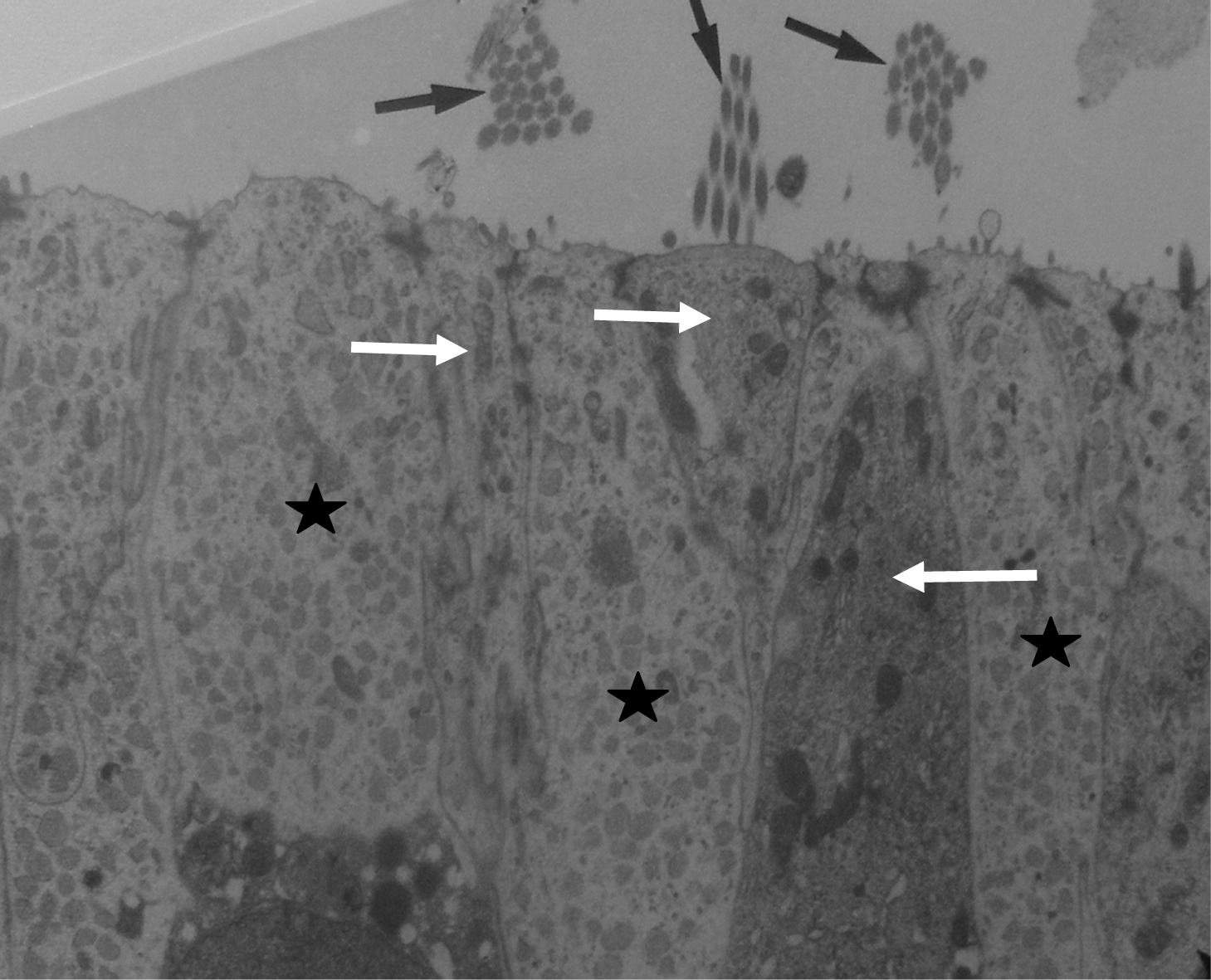

Supplement: Figure S3 — Electron microscope image of sensory tissue below the cupula. Sensory cells (white arrows) with hairbundles (black arrows) are shown, adjacent to supporting cells (asterix). (TIF) [file pone.0111917.s003.tif]
